# Iron Redox Chemistry and Implications in the Parkinson's Disease Brain

**DOI:** 10.1155/2019/4609702

**Published:** 2019-10-09

**Authors:** Dinendra L. Abeyawardhane, Heather R. Lucas

**Affiliations:** Department of Chemistry, Virginia Commonwealth University, Richmond, VA 23284, USA

## Abstract

The etiology of Parkinson's disease (PD) is linked with cellular inclusions in the *substantia nigra pars compacta* region of the brain that are enriched in the misfolded presynaptic protein *α*-synuclein (*α*S) and death of the dopaminergic neurons. Brain iron homeostasis governs both neurotransmission and neurodegeneration; hence, the role of iron in PD progression and neuronal health is apparent. Elevated iron deposits become prevalent in the cerebral region upon aging and even more so in the PD brain. Structural as well as oxidative modifications can result from coordination of *α*S with redox active iron, which could have functional and/or pathological implications. In this review, we will discuss iron-mediated *α*S aggregation, alterations in iron metabolism, and the role of the iron-dopamine couple. Moreover, iron interactions with N-terminally acetylated *α*S, the physiologically relevant form of the human protein, will be addressed to shed light on the current understanding of protein dynamics and the physiological environment in the disease state. Oxidative pathways and biochemical alterations resulting from aberrant iron-induced chemistry are the principal focus of this review in order to highlight the plethora of research that has uncovered this emerging dichotomy of iron playing both functional and disruptive roles in PD pathology.

## 1. Introduction

Parkinson's disease (PD) is identified as the second most prevalent neurodegenerative disorder in the world, and it is an age-related progressive disease [1]. It has been estimated that approximately 1 million people in the United States alone are suffering from PD, where the majority of that population is over the age of 60. The key symptoms of PD include resting tremors, slow movements, muscle stiffness, and difficulties performing voluntary movements [2]. The main characteristic feature associated with PD is the loss of dopaminergic neurons in the *substantia nigra pars compacta* (SNpc), and these neurons are the principal source of dopamine (DA), a neurotransmitter that regulates motor behavior [3]. PD etiology is also linked to the presence of cellular inclusions, known as Lewy bodies (LBs), which have been identified as postmortem hallmarks of PD [4, 5]. The major constituent of LBs is aggregated *α*-synuclein (*α*S), an intrinsically unfolded protein predominantly localized in the presynaptic regions of dopaminergic neurons. A tetrameric form of *α*S has also been identified in human tissues that has been postulated as the inactive storage form of this structurally dynamic protein due to its aggregation resistance [6, 7]. Although the cellular mechanisms and normal functions associated with *α*S have not yet been completely resolved, the increased levels of *α*S in the brains of patients with PD suggest a relationship with the neurotransmitter DA [8].

**Figure 1:**
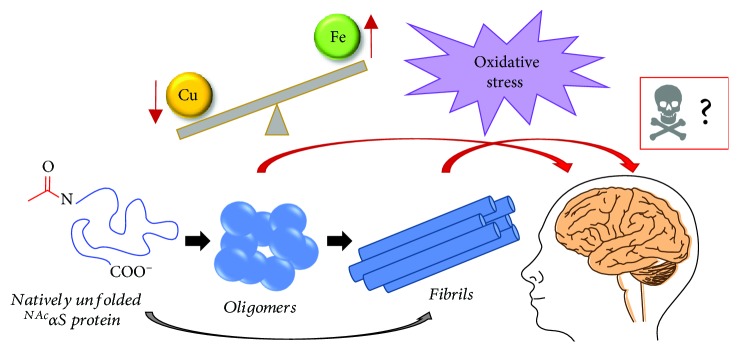
Schematic diagram of *α*S aggregation.

It is well known that metal homeostasis plays an important role in regulating cellular functions [9]. Copper, iron, manganese, and zinc are essential transition metal ions for neurotransmission, enzymatic reactions, and mitochondrial functions regulated by the central nervous system (CNS) [10]. Thus, an imbalance of metal ions has an impact on disease states, among which are neurodegenerative disorders. *In vivo* magnetic resonance imaging (MRI) has highlighted the accumulation of iron in the SNpc region of the PD brain, which disrupts iron circulation pathways to create an imbalance of the metal [11, 12]. In a separate study, meta-analysis of literature data on blood serum iron levels also pinpointed an increase in this metal [13]. However, a direct correlation between iron deposition and iron transportation within the brain has not been established in PD patients [14]. Beyond PD, *in vivo* MRI mapping of iron content among neurodegenerative tremulous diseases has also indicated a deposition of iron in the SNpc as compared to healthy controls [15, 16]. It is worth noting that iron dyshomeostasis associated with neurodegeneration is influenced by age, race, and gender. Therefore, iron-sensitive MRI mapping could be a powerful tool to diagnose and differentiate neurodegenerative diseases at an early stage. Evidence for iron accumulation within the SNpc region of the PD brain along with abundant misfolded *α*S inclusions implies a direct relationship between iron and *α*S in the pathogenesis of PD [17]. As shown in Figure 1, *α*S found in LBs exists in the form of highly ordered aggregated species that can be described as oligomers and/or fibrils. Under an oxidatively stressed environment, the aggregation processes of *α*S are majorly affected by interaction with redox active iron [18].

In this review, structural and biochemical consequences associated with iron-bound *α*S and alterations in the intracellular iron composition within dopaminergic neurons will be discussed. Key roles of iron will be delineated with respect to both the healthy brain and the PD brain. Cerebral iron levels are often associated with the modulation of dopamine-related biochemical pathways in the brain; therefore, the impact of the iron-dopamine couple and the disruption of biological cascades associated with these two components will be addressed in this review. Moreover, the impact of iron redox chemistry on the *α*S structure will also be highlighted.

## 2. *α*-Synuclein Structure and Iron Coordination

**Figure 2:**
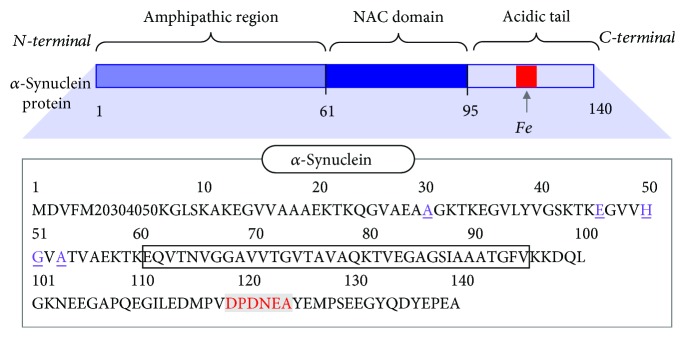
Cartoon representation of the N-terminal region, NAC region (highlighted within the box), and C-terminal region of *α*S with the primary amino acid sequence shown underneath. The iron binding site is highlighted at the C-terminal region in red text; genetic PD mutations are colored and underlined in purple.

*α*S, encoded by the SNCA gene, consists of 140 amino acids and contributes approximately 1% of the protein content in cytosol [4, 5]. There are three distinct regions of *α*S protein (Figure 2). The N-terminal region, which contains 11 imperfect amino acid repeats with a consensus sequence KTKEGV, adopts an amphipathic *α*-helix conformation upon membrane interaction [8, 19]. The central non-amyloid-*β* component (NAC region) is involved in aggregation pathways and contains the hydrophobic core of *α*S. The C-terminal region is an acidic segment rich in negatively charged amino acid residues, namely, aspartic acid (D) and glutamic acid (E), and is highly dynamic. Although the pathogenesis of sporadic PD is mainly driven by the vulnerability of dopaminergic neurons, several genetic point mutations in *α*S have been identified as associated with PD, including A30P, E46K, H50Q, G51D, A53E, and A53T (mutation sites are highlighted in Figure 2) [20]. In fact, enhanced aggregation properties have been reported for E46K, H50Q, and A53T as compared to wild-type, suggesting that these single point mutations initiate dramatic structural changes that seed fibrillation. The familial mutant A53T has also been reported to possess increased neurotoxicity upon elevated iron levels in comparison to wild-type *α*S due to selective loss of DA neurons as well as motor impairment [21].

Postmortem analyses of brain tissues isolated from patients with PD or dementia with Lewy bodies (DLB) have indicated that *α*S is acetylated at the N-terminus [22, 23]. Thus, it has been confirmed that N-terminally acetylated *α*S is the physiologically relevant form of the protein [24]. N-terminal acetylation is the transfer of an acetyl group from acetyl coenzyme A to the *α*-amino group of the first amino acid residue of a protein, as in methionine (M) for *α*S. Although it is a common posttranslational modification catalyzed by different N-terminal acetyltransferases (NATs) in that approximately 84% of human-derived proteins carry this modification [25–27], the majority of *α*S research has been carried out with the nonacetylated *α*S variant. The consequences of this modification are becoming increasingly more apparent with respect to membrane interactions, metal coordination, and the protein folding tendencies [18, 28–31]. The innate structural dynamics and behavioral patterns of *α*S are strongly influenced by N-terminal capping; for example, this modification can further stabilize the tetrameric structural orientation of *α*S [7].

The inherent metal binding properties of *α*S have been vastly studied [9, 10, 32, 33]. Among the prevalent redox active transition metals in the brain, copper has shown the highest binding affinity to *α*S [34]. Copper levels are diminished in the PD brain, while iron levels are elevated [35]. Thus, iron-*α*S interactions would be more prevalent under disease conditions. The primary binding site of iron at the C-terminus, ^119^DPDNEA^124^ (Figure 2), is a motif rich in negatively charged amino acids that will facilitate hard acid/hard base interactions via potential iron-oxygen coupling. The presence of a proline residue can also facilitate protein folding dynamics to form a more stable global configuration upon iron binding. It can also be postulated that binding of iron at the C-terminus of *α*S (pI of 4.7) causes a neutralization of the negative charges [36]. Such charge pairing can result in electrostatic shielding, which may impact protein folding dynamics by further altering the structure upon protein oxidation, crosslinking, aggregation, etc.

Notably, the metal coordination sites for iron and copper are located at opposite termini, where iron preferentially interacts within the *α*S C-terminal region, and copper has two separate sites within the N-terminus that are dependent on the copper redox state. Davies et al. have shown that there are only subtle changes in the binding affinity upon iron(III) interaction with copper(II)-saturated *α*S, indicating that the binding of the two metals is independent from each other [37]. Copper was further proposed as a cofactor for *α*S due to the measured ferrireductase activity in the presence of an electron accepter such as NADH [37].

The ability of *α*S to transform iron(III) to iron(II) highlights a ferrireductase activity of *α*S that could uncover a functional role. Conversely, the overexpression of *α*S could lead to excessive generation of iron(II) that may eventually result in an oxidative stress environment due to reactive oxygen species (ROS) production mediated by the Fenton reaction. Recently, we also reported evidence to support the ferrireductase activity of *α*S upon binding to iron(III) under anaerobic conditions as well as an increase in the antiparallel *β*-sheet composition as is characteristic of *α*S aggregates formed under oxidizing conditions in the presence of Fe^II^ [18]. On a similar note, Ortega and coworkers have reported that intracellular overexpression of *α*S in neurons promotes the accumulation of iron in the perinuclear region [38], accentuating that iron binding to *α*S correlates with *α*S aggregation and iron deposition as is reported for PD. Detection of iron in these regions could be acknowledged as an indirect biomarker for PD.

## 3. Iron-Mediated *α*-Synuclein Aggregation

The typical *α*S aggregation pathway involves propagation of natively unfolded monomers to higher-ordered oligomeric species which eventually form fibrillar structures [39, 40]. Specifically, the cardinal conformational alteration occurs when the disordered, “random coil” structure of the native protein is transformed into the well-known “*β*-sheet” structure concentrated in both oligomeric and fibrillar macromolecular aggregates. The orientation patterns of *β*-sheet (parallel or antiparallel) are distinguishable within certain contexts [41]. Although the *in vivo* toxic form of aggregates is not yet fully identified, antiparallel *β*-sheets are believed to be a characteristic of the toxic form [41]. The least compact oligomeric intermediates are reported to be toxic due to disruptive cellular functions including cellular leakages generated by membrane pores [42]. Hence, a correlation between oligomers and antiparallel *β*-sheets is expected. Based on nuclear magnetic resonance (NMR) spectroscopy and high-resolution cryoelectron microscopy techniques, the fold of fibrils is proposed to be parallel *β*-sheets [43]; however, the antiparallel versus parallel conformations of *α*S oligomers and/or fibrils have not yet been fully discerned. Traditionally, the mechanistic folding pathway is thought to involve an oligomer state that precedes fibrillation [44]; however, off-pathway aggregation routes have also been reported [18]. Fibrillation pathways via oligomer intermediates have also been thought to progress from antiparallel to parallel *β*-sheets [45].

The redox activity of metals like copper and iron is linked with oxidative and/or nitrosative stress and contributes as a major factor to the aggregation of *α*S [18, 30, 31, 46, 47]. For example, rat neuronal cultures rich in *α*S aggregates have indicated excessive free radical formation, implying oligomer-induced oxidative stress which is also metal-dependent [48]. It has also been suggested that iron(III)-induced SDS-resistant oligomers of *α*S form pores in the lipid planar bilayer in the presence of ethanol or DMSO, which eventually lead to toxicity as a result of permeability [49, 50]. Previously, it was reported that fibrils generated over the course of aggregation with iron(III) adopt a distinctly different morphology when compared to analogous fibrils with Cu(II) [36]. Transition electron microscopy (TEM) analysis of the stoichiometric Fe^III^-*α*S aggregates indicated the presence of a short, thick, network-like fibrillar structure without any amorphous material. Growth patterns and morphology of fibrils are a potential biomarker which can aid in understanding structural factors affecting cell viability in neurodegeneration.

Oligomer formation can be coupled with potential membrane interactions related to pore-forming proteins which regulate metal homeostasis [49]. Oligomers generated in the presence of both iron(III) and iron(II) have also been suggested as nonharmful and to instead possess a potential functional role [51–53]. The Xie group has reported that the aggregation of *α*S induced by iron is dose- and time-dependent [54]. The cell viability assay conducted with SK-N-SH human neuroblastoma cells indicated that the higher the iron concentration (>1 mmol/L) and longer the period of aggregation (>24 h), the more toxicity to cells [54, 55]. Diminished mitochondrial transmembrane potential and elevated ROS production indicated the pivotal role of iron in inducing cytotoxicity. Cell toxicity due to iron-induced *α*S aggregation was treated by silencing the intracellular expression of *α*S using siRNA [56]. As it is evident that iron-mediated oxidative stress leads to cytotoxicity, it will be beneficial to look into iron chelators (Section 6 in this review), which could attenuate disease progression.

**Figure 3:**
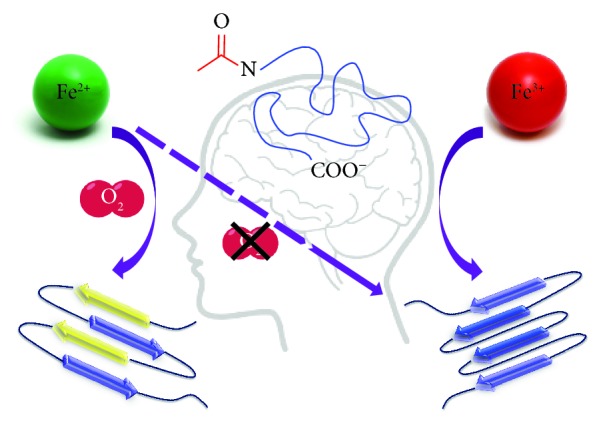
Graphical representation of N-terminally acetylated *α*S structural consequences upon aggregation mediated by iron-oxygen chemistry.

In order to shed some light on the iron-*α*S interaction, our group focused on iron-mediated aggregation of *α*S, marking the first publication to address the relationship of the iron-*α*S couple on the native N-terminally acetylated form of the protein [18]. A distinct change in the protein conformation was noted demonstrating the iron-oxygen-driven generation of an oligomer-locked iron-*α*S structure rich in right twisted antiparallel *β*-sheets (Figure 3). The PD relevance of this oligomeric motif was determined by its positive response to the anti-oligomer A11 polyclonal antibody, which selectively identifies soluble oligomeric epitopes present in common amyloidogenic proteins such as *α*S (PD), amyloid-*β* (Alzheimer's disease), IAPP (type II diabetes), and prion protein (Creutzfeldt-Jakob disease or mad cow disease) [57]. Hence, our results highlight the major role of iron redox chemistry in the process of *α*S oligomerization.

## 4. Iron Metabolism and Alterations in the PD Brain

**Figure 4:**
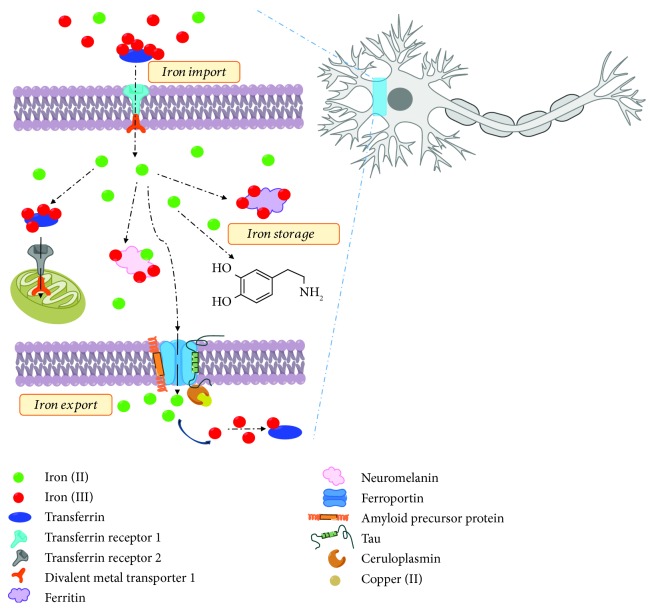
Iron metabolism in the neuron.

Iron plays a functional role in brain biochemistry by acting as a cofactor for tyrosine hydroxylase (TyrH), an enzyme that initiates the conversion of tyrosine to DA in the cytosol [58, 59]. Iron additionally serves as an essential element in various fundamental processes within the CNS, including mitochondrial respiration, DNA synthesis, myelin production, neurotransmission, and metabolism [60]. The innate redox nature of iron is coupled with electron transfer processes. Hence, the redox state of iron, whether ferrous (Fe^2+^) or ferric (Fe^3+^) ions, governs the feasibility of various iron-dependent biological functions. Dysfunction or imbalance of the equilibrium between iron(II) and iron(III) ions can disrupt processes due to the generation of ROS as is commonly associated with Fenton chemistry. Therefore, iron homeostasis plays a pivotal role in regulating cellular functions as is briefly illustrated in Figure 4.

Brain iron uptake is mainly driven by the glycoprotein transferrin (Tf), the primary iron transport protein in the CNS [61, 62]. Studies have shown that non-transferrin-bound iron levels are high in the cerebrospinal fluid due to controlled Tf transportation through the blood-brain barrier [63]. Tf possesses high affinity iron(III)-binding sites, and transferrin-bound ferric ions are engulfed into cells with the aid of transferrin receptor-1 (TfR-1) via endocytosis [62]. Newly imported ferric ions are subsequently reduced to ferrous ions and released into the cytosol by divalent metal transporter-1 (DMT-1). Intracellular iron levels are further controlled by iron regulatory proteins (IRP) that act in concert with DMT-1 and TfR-1. In iron-deficient cells, IRPs selectively bind to an iron responsive element (IRE) that facilitates iron uptake by stabilizing the mRNA coding for TfR-1 and DMT-1 [64]. IRE adopts a loop-like structure consisting of 26-30 nucleotides, often present in 3′ or 5′ untranslated regions (3′-UTR or 5′-UTR) of eukaryotic mRNA for iron-dependent translational control. Another iron transporter, transferrin receptor-2 (TfR-2), which does not have an IRE, is also found in the dopaminergic neurons of the SNpc and concentrated more within the mitochondria of these cells [65]. Mitochondrial dysfunction has been accompanied by elevated Tf and TfR-2 levels in PD, suggesting oxidative stress promoted by iron redox chemistry [17, 62, 65].

Translation of ferritin, the main iron storage protein in the body, is also regulated by the availability of intracellular iron, as an IRE is found in the 5′-UTR of Tf mRNA [66]. Interestingly, a region in the 5′-UTR of human *α*S mRNA is reported to possess a high resemblance to the IRE present in Tf mRNA [67]. A potential IRE motif in *α*S mRNA suggests the possibility of iron-dependent posttranscriptional regulation of *α*S protein generation. Polysomal RNA analysis conducted after treatment with iron chelators has confirmed that expression of *α*S is influenced at a translational level by iron availability [66]. Another essential iron storage macromolecule in neurons, neuromelanin, has also been detected to accumulate in the SNpc of PD patients [68]. Both ferritin and neuromelanin possess dense iron cores [69, 70]. In the dopaminergic neurons, the ferric ions are easily reduced to ferrous ions by cytotoxic by-products of DA oxidation [17, 58, 59, 62, 71]. The ability of some cellular components, such as melanin, to reduce iron(III) to iron(II) can stimulate the hydroxyl radical formation mediated by Fenton chemistry [61, 69].

Physiological iron levels are expected to rise with aging; however, a drastic elevation is noted in PD patients [72]. Pathological iron dyshomeostasis affects the progression of PD, resulting from cumulative events that affect the capacity for neuronal survival. Suppressed expression of ferroportin and increased expression of DMT-1 mainly contribute to the elevated levels of iron in the body. Ferroportin, an iron efflux pore, is predominantly responsible for neuronal iron export, yet the efficiency of ferroportin is not governed by cellular iron levels alone [62]. There are several additional factors contributing to inefficient iron export from the neurons. For example, hepcidin is an iron regulatory hormone responsive to iron overload and inflammation. Binding of hepcidin to surface ferroportin impedes iron export via cellular internalization and degradation of ferroportin [73]. The ferroxidase activity of ceruloplasmin, a multicopper oxidase enzyme, can facilitate iron efflux coupled to ferroportin, enabling iron(III) to become readily available for binding with extracellular transferrin [74]. Notably, the low levels of copper in the SNpc as has been reported in PD patients correlate with low activity of ceruloplasmin, thus contributing to the intracellular accumulation of iron [17, 35, 75]. In addition, tau protein interacts with amyloid precursor protein to promote ferroportin-mediated iron export, and reduced levels of both of these neuronal proteins have been reported in PD brains [76]. Hence, the consequences of iron accumulation collectively disrupt cellular pathways that are dependent on our metabolism. Disruption of intracellular homeostasis could potentially be utilized to develop an indirect set of biomarkers for the diagnosis of PD based on various cellular components and proteins that are less commonly linked to PD. However, quantitative statistical analyses based upon external variables, such as age and ethnicity, have to be taken into account to establish a standard biomarker identification system.

## 5. Toxic Consequences of the Interplay between Iron and Dopamine

**Figure 5:**
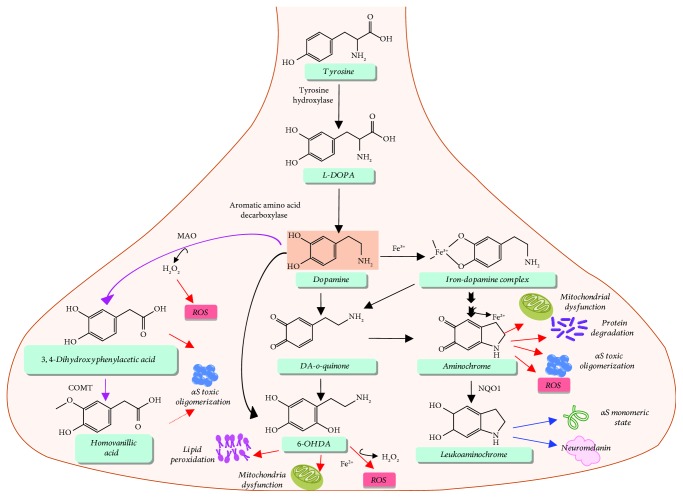
Schematic representation of dopamine metabolism in the synapse and toxic cascades associated with iron.

Biosynthesis of DA is initiated from the amino acid tyrosine following its import into dopaminergic neurons by amino acid transporters (Figure 5) [77]. The rate-limiting step of DA synthesis is the conversion of tyrosine to dihydroxyphenylalanine (L-DOPA), which is driven by TyrH [77]. The active site of TyrH requires an iron atom in its ferrous form as a cofactor; thus, a deficiency of ferrous ions debilitates DA synthesis [77, 78]. The subsequent conversion of L-DOPA to DA is driven by aromatic amino acid decarboxylase (AADC, dopa decarboxylase), and it is selective to L-amino acid substrates [77]. Due to the dependency of the DA metabolic pathway on iron, changes in the redox state balance of Fe(III)/Fe(II) in PD brains and/or alterations in the iron flux can directly impact the health of dopaminergic neurons.

The major metabolites of enzymatic DA degradation are 3,4-dihydroxyphenylacetic acid and homovanillic acid (Figure 5) [79, 80]. DA degradation is prompted by monoamine oxidase (MAO) enzyme which results in 3,4-dihydroxyphenylacetaldehyde and hydrogen peroxide. Both substances are highly reactive and potential candidates for neurotoxicity. Under physiological conditions, 3,4-dihydroxyphenylacetaldehyde is readily oxidized to 3,4-dihydroxyphenylacetic acid, eventually leading to formation of homovanillic acid via catechol-*o*-methyl transferase (COMT) [79]. Elevated levels of both of these metabolites are identified in cerebrospinal fluid in patients with motor disorders or early PD symptoms. Hence, clinical studies emphasize that 3,4-dihydroxyphenylacetic acid and homovanillic acid could be pivotal biomarkers in PD progression [81]. In particular, the highly reactive nature of 3,4-dihydroxyphenylacetaldehyde can initiate hydroxyl radical generation leading to *in vitro* and *in vivo* neurotoxicity [82]. In fact, immunoanalyses have demonstrated that aggregation of *α*S both *in vitro* and *in vivo* is enhanced by 3,4-dihydroxyphenylacetaldehyde in a dose-dependent manner [83]. Furthermore, the aggregation is provoked by this metabolite to potentially form toxic oligomers [83].

In the presence of ferric ions, DA undergoes oxidation to generate DA-*o*-quinone (Figure 5), which can enter into neurotoxic pathways and eventually promote degeneration of dopaminergic neurons [84]. DA oxidation can be governed by several factors, such as oxygen, inorganic reagents, and redox active metals (primarily manganese, copper, and iron) [85–89]. Iron-facilitated DA oxidation forms another neurotoxic byproduct called 6-hydroxydopamine (6-OHDA) [90, 91], which perturbs mitochondrial functions and consequently promotes acute cell death due to disruption in ATP synthesis [92]. It also contributes to oxidative stress by producing H_2_O_2_, eventually triggering lipid peroxidation and cell apoptosis [92].

Conversely, DA oxidation is also an essential step in the synthesis of neuromelanin [84], which is rapidly generated in the presence of iron(III) via formation of an iron-DA complex (Figure 5). While promoting biological reactions, the redox chemistry of iron can simultaneously influence the ROS and reactive nitrosative species (RNS) formation leading to lipid peroxidation, DNA/protein degradation, and ultimately cell death. Neurotoxicity coupled with the iron-DA complex depends upon its cellular uptake. The stable precursor of neuromelanin synthesis, aminochrome, can lead to subsequent ROS generation mediated by Fenton reactions and *α*S aggregation [93–95]. In addition, aminochrome can progress mitochondrial dysfunction, protein degradation, and oxidative stress in the neurons [96–98]. Toxic oligomerization of *α*S associated with aminochrome can be prevented by DT-diaphorase (NQO1), a flavoenzyme involved in the quinone reduction pathways [93]. This enzyme attenuated aggregation by stabilizing the monomeric state of *α*S upon catalyzing the reduction of quinone (aminochrome) to leukoaminochrome. In a separate study, the fibrillization pathway of *α*S has also been altered to spherical oligomer formation following DA oxidation [94]. The function of *α*S is also believed to be associated with DA metabolism [99]. DA interaction with *α*S leads to formation of SDS-resistant soluble oligomeric species upon oxidation of methionine residues in the *α*S amino acid sequence (Met locations: 1, 5, 116, and 127), consequently thwarting *α*S fibrillation [100, 101]. Modulation of aggregation pathways associated with *α*S by DA causes an increase in *α*S oligomers in the extracellular environment. Even though the disruptive mechanism behind the oligomerization is still under debate, these soluble oligomers are believed to be neurotoxic. Hence, a more detailed analysis of *in vivo* oligomerization and its potential to form cytotoxic membrane pores will be essential to understand the pathophysiology of the disease state.

## 6. Inhibition of Iron-Mediated Aggregation

Iron accumulation in the brain is implicated in diseases beyond PD, including rare synucleinopathies such as neurodegeneration with brain iron accumulation type 1 (NBIA1), multiple system atrophy (MSA), essential tremor (ET), progressive supranuclear palsy (PSP), and tremor in dystonia [12, 16, 102]. As aforementioned, increasing evidence supports the fact that the aggregation propensity of *α*S is affected by the redox activity of iron, and exposure to iron and oxygen stimulates a likely toxic oligomeric form of *α*S [18]. Therefore, inhibition of aggregation and iron chelation as a prevention technique has been briefly addressed in this section.

**Figure 6:**
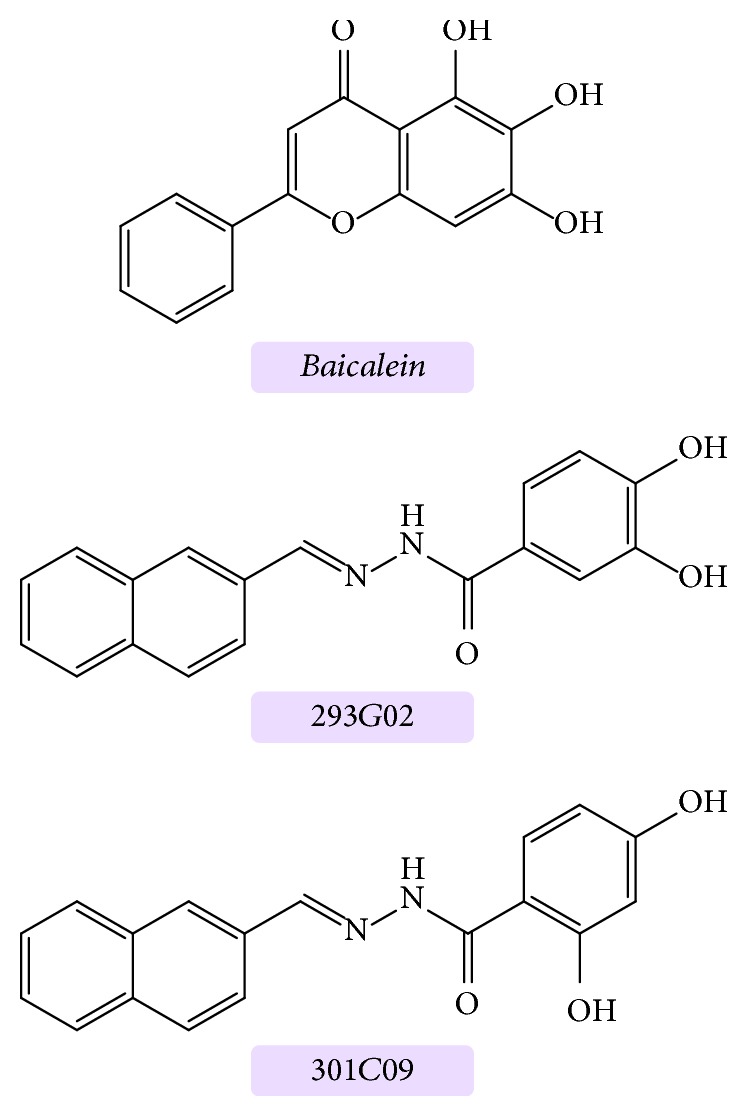
Structures of inhibiting agents.

Baicalein and N′-benzylidene-benzohydrazide (NBB) derivatives, such as 293G02 and 301C09, have been shown to inhibit iron-induced oligomer formation and/or fibrillation (Figure 6). Low concentrations of baicalein have proven to be an excellent candidate to block the aggregation of *α*S initiated by treatment with organic solvent [49]. The catechol moiety of NBB derivatives governs the inhibitory activity, highlighting a key structure-activity relationship [103, 104]. In particular, the compound 293G02 was highly active at controlling oligomerization inhibition, with cytotoxicity assays indicating a significant reduction in toxicity [104].

Aggregation promoted by iron can also be affected by the presence of redox inactive metal ions under physiological conditions. For example, Golts and coworkers have reported that iron-mediated *α*S aggregation can be inhibited by the presence of magnesium(II) by negatively modulating the iron(II) affinity [105]. It was suggested that an altered conformation enabled resistance to aggregation rather than competing with the same iron coordination site. Therefore, other metals that can compete for the same binding site in *α*S could act as potential therapeutic agents by mitigating the harmful effect of iron-promoted oligomerization.

Exposure of iron at early stages of life has been identified as a potential risk factor of PD [106]; however, the toxicity imparted upon early exposure to iron is irreversible even in the presence of a moderate chelator. Hence, the iron-*α*S interaction window is critical in PD progression as well as in the clinical aspects of disease prevention. Treatment with an iron chelator such as clioquinol has been shown to reduce nigral iron resulting in an increase in the cell viability [107]. In a separate *in vivo* study, treatment of iron-induced *α*S aggregates with deferiprone, a ferric ion chelator, has displayed improved motor functions in mouse models implying that the clinical application of iron chelation holds promise [108]. Structure-activity relationships among iron chelators will require careful attention as a means to open up new avenues in neurodegenerative drug discovery.

## 7. Concluding Remarks

Brain iron dyshomeostasis plays a crucial role in neurodegeneration associated with PD. Oxidative and conformational modifications of *α*S have a clear link to PD etiology, designating this structurally dynamic protein as a major target for therapeutic studies among the research community. The involvement of iron with *α*S biochemistry has been studied less extensively in comparison to research on copper-*α*S interactions; however, many studies have begun to address the potential structural and oxidative consequences that lead to *α*S deposits as a result of iron accumulation in the PD brain. Iron regulation in neurons has already shed light into clinical applications, and new research highlighted in this review may provide an avenue towards future therapeutic studies and/or inspire new biomarkers for PD.
